# Factors influencing mammalian community occupancy in Dhorpatan Hunting Reserve, Nepal

**DOI:** 10.1002/ece3.9980

**Published:** 2023-04-07

**Authors:** Sandeep Regmi, Jerrold L. Belant, Bindu Pant, Hari Prasad Sharma

**Affiliations:** ^1^ Central Department of Zoology, Institute of Science and Technology Tribhuvan University Kathmandu Nepal; ^2^ Department of Fisheries and Wildlife Michigan State University East Lansing Michigan USA; ^3^ Nepal Zoological Society Kathmandu Nepal

**Keywords:** mammals, modeling, multi‐species, occupancy, threats

## Abstract

The decline in mammalian species diversity is increasing worldwide, with areas characterized by high human activities experiencing more prominent effects. Knowledge of spatial distributions of species and factors acting on them is necessary for effective management. We evaluated community‐level occupancy of mammal species in Dhorpatan Hunting Reserve (DHR), Nepal using remote cameras during 15 March–15 June 2022. We used mammal species detections from remote cameras and multispecies hierarchical occupancy modeling to assess the effects of environmental and anthropogenic variables on community‐level occupancy of detected mammal species. We identified a highly heterogeneous mammal species community at DHR with greatest detection probability (0.21) for red fox (*Vulpes vulpes*) and lowest (0.08) for blue sheep (*Pseudois nayaur*). Naïve occupancy ranged from 0.31 for giant‐flying squirrel (*Petaurista magnificus*) to 0.84 for red fox. Mammal community occupancy increased with increasing canopy cover and number of livestock detections, but overall occupancy declined close to human settlements. The findings of this study can be used for developing policy at DHR for the management of mammal species through reducing the potential increase of human settlements or livestock grazing.

## Graphical abstract

Mammal community occupancy increased with increasing canopy cover and a number of livestock detections, but overall occupancy declined nearer to human settlements.

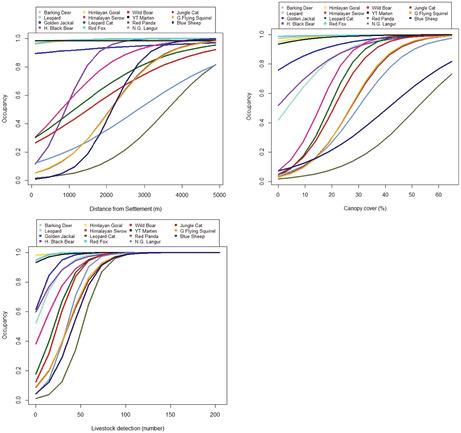

## 1 INTRODUCTION

Knowledge of species distributions and factors that influence them is a crucial part of ecology and conservation (Gotelli & Colwell, 2001). Recent anthropogenic effects on species' habitats have increased and consequently reduced global biodiversity (Drouilly et al., 2018). These anthropogenic factors impact species occurrences and distributions, which can vary spatially and among species (Karanth et al., 2009; Leweri et al., 2022). Thus, there is a need to monitor and manage species' habitats in response to these threats (Drouilly et al., 2018; Kalle et al., 2013; Leweri et al., 2022). However, most studies are focused on species‐level management (Carroll et al., 2001; Epps et al., 2011; Lambeck, 1997), whereas a community‐level approach can provide greater information, including knowledge of species interactions (Simberloff, 1998; Wiens et al., 2008).

Community‐level information can be gained using multispecies approaches including occupancy modeling, which uses species presence data to gain inferences about their distributions (Balmford et al., 2005; Dorazio & Royle, 2005; Yoccoz et al., 2001). Species‐ and community‐level occupancy is determined by some set of variables (Shmida & Wilson, 1985) which can include anthropogenic factors like settlements, roads, and livestock. For example, mammal community occupancy in China decreased with increasing cattle detections and vehicle traffic but increased with distance to settlement, human detections, and plant productivity (Feng et al., 2021). Similarly in the rangelands of Karoo, South Africa, mammal and ground bird species occupancy increased with increasing livestock detections but decreased with increasing human presence (Drouilly et al., 2018). Environmental factors like canopy cover can also influence occupancy probability of species (Laurance et al., 2008; Whitworth et al., 2019). In Manu Biosphere Reserve, Peru, rainforest mammal species occupancy increased with greater canopy cover and decreased with increasing forest disturbance (Whitworth et al., 2019). Similar effects of canopy cover on species occupancy occurred with nocturnal mammals in African rainforests (Laurance et al., 2008) and mammalian carnivores in Brazil (Regolin et al., 2017).

Under high anthropogenic activities, the probability of encounter between humans and wildlife becomes higher, which leads to conflicts between them (Treves & Karanth, 2003). In these areas, coexistence between wildlife and human should be maintained for their long‐term conservation. Assessing animal abundance is prerequisite for their effective management and wildlife conservation (Blanc et al., 2007). For effective assessment, observations of animals should be combined with inferential methods including the potential influence of considered variables (Dorazio & Royle, 2005; MacKenzie & Kendall, 2002; Yoccoz et al., 2001), and these inferences work efficiently only if the system dynamics are generated by manipulative experiments (Fisher et al., 1943; Hurlbert, 1984; Pianka, 1966). Recent advancements in camera‐based studies on multiple species have emphasized particular species guilds (Schuette et al., 2013; Stoner et al., 2007), with few efforts to investigate entire communities (Rich et al., 2016), highlighting need to study community‐level occupancy. In Nepal, studies of species occupancy have mostly focused on single species (Barber‐Meyer et al., 2013; Lamichhane et al., 2021; Sharma et al., 2020; Thapa et al., 2020; Thapa & Kelly, 2017).

Dhorpatan Hunting Reserve (DHR) in Nepal is a highland protected area with human access for natural resource use, which can increase adverse effects between humans and wildlife. However, there have been no investigations of mammalian community response to human activities in DHR. We quantified multispecies occupancy of mammal species and the impacts of habitat variables on their occupancy in DHR. Due to the potential impacts of anthropogenic activities in DHR, we predicted that occupancy of wild mammal species in DHR would be adversely affected by anthropogenic factors such as human settlements and livestock.

## 2 MATERIALS AND METHODS

### 2.1 Study area

**FIGURE 1:**
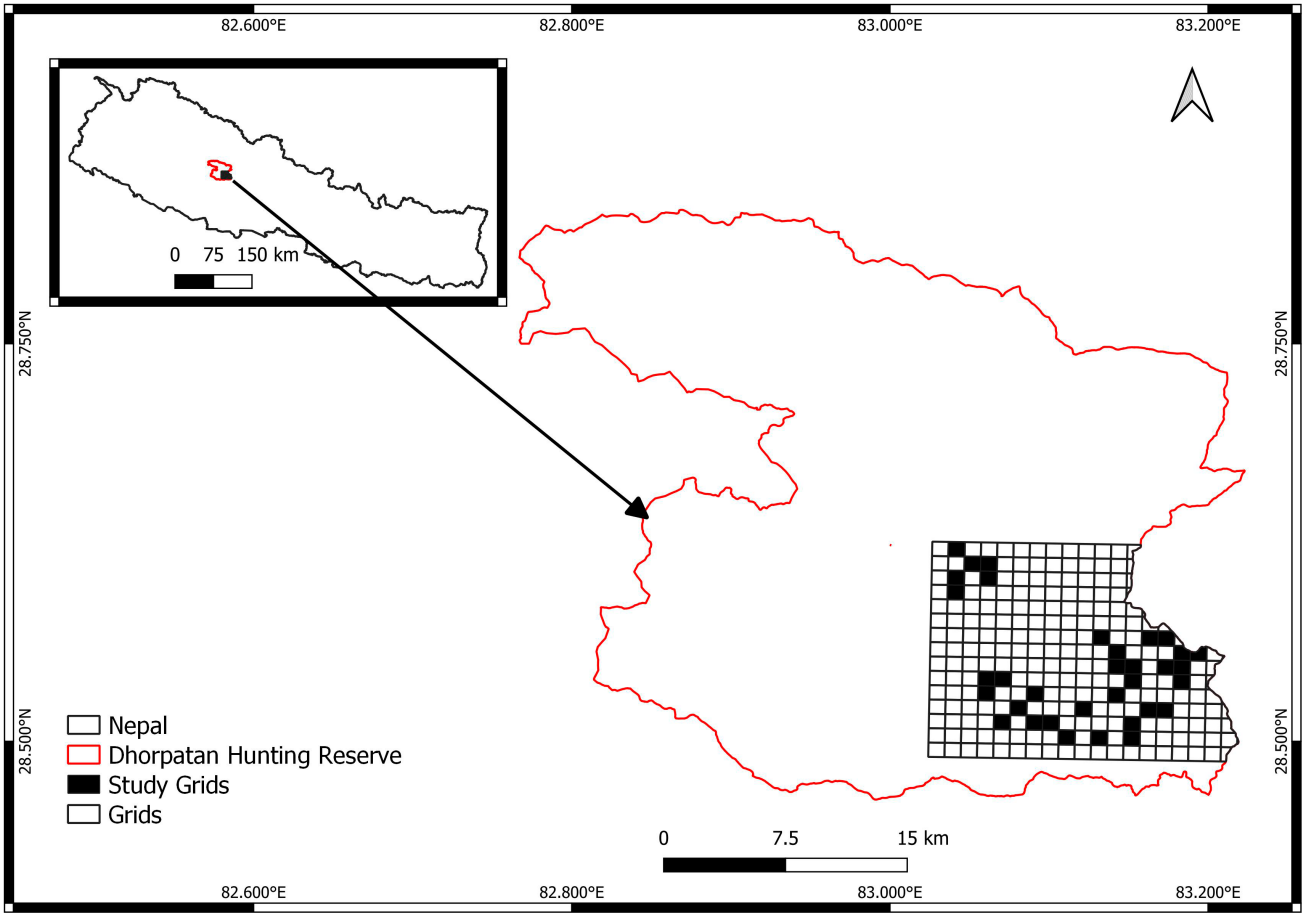
Grid of 1‐km^2^ cells for deploying remote cameras, Dhorpatan Hunting Reserve, Nepal, 2022.

Dhorpatan Hunting Reserve (28°33′20″–28°48′00″N and 82°51′00″–83°12′00″E) comprises 1325 km^2^ in the Rukum, Myagdi, and Baglung districts of the Dhawalagiri Mountains (Figure 1) and is the only hunting reserve for blue sheep (*Pseudois nayaur*) and Himalayan tahr (*Hemitragus jemlachicus*) in Nepal (DHR, 2019). The reserve is adjacent to villages and settlements except along the northern boundary. Elevations are 3000–7000 m above sea level, with flat meadows above 4000 m. Temperatures range from an average low of 1.4°C in winter to an average high of 24.8°C in summer.

The reserve contains alpine, subalpine, and high‐temperate vegetation with mixed‐hardwood forests including fir (*Abies spectabilis*), pine (*Pinus roxburghii*), birch (*Betula utilis*), Rhododendron (*Rhododendron arboretum*), hemlock (*Tsuga dumosa*), oak (*Quercus leucotrichophora*), junipers (*Juniperus recurva*, *J. indica*), and spruce (*Picea smithiana*). At higher elevations, more than 50% of the reserve consists of pastures. Faunal diversity of DHR includes 32 mammal species such as snow leopard (*Panthera uncia*), barking deer (*Munticus vaginalis*), blue sheep, leopard (*Panthera pardus*), Himalayan goral (*Naemorhedus goral*), Himalayan tahr (*Hemitragus jemlachicus*), black bear (*Ursus thibetanus*), red panda (*Ailurus fulgens*), rhesus macaque (*Macaca mulatta*), Himalayan serow, wild boar (*Sus scorfa*), and gray wolf (*Canis lupus*).

### 2.2 Data collection

For identifying species presence, we deployed remote cameras in Fagune and Barse blocks of DHR during 15 March–15 June 2022. We first created a grid of 290 plots 1 km^2^ cells for camera deployment. We deployed a single camera (Stealth Cam STC‐G45NG) in each of 34 randomly selected cells (116 km^2^ overall area), with a spacing of about 1 km between cameras in adjacent cells. We placed cameras 50–100 cm above ground and typically near trails, water bodies, or other areas where species were likely to be detected. We set cameras to take three images for each detection with a 30‐s delay. This study took each 14 days survey period as a single sampling occasion across the study.

We measured canopy cover, distance to nearest human settlement, and number of times livestock were detected at each camera site. We recorded forest canopy cover using Gap Light Analysis Mobile Application (GLAMA; Tichý, 2016) in each camera trap location. We established a 10 × 10 m plot at the center of each camera trap location and measured the canopy cover from four corners and the center of each plot. We measured distance to nearest human settlement using a measuring tape if less than 200 m and Google Earth when the distance exceeded 200 m. We determined the number of times livestock were detected using images from each camera. Because large herds generally occur in DHR, we counted sequential detections (at 30‐s intervals) as a single detection when it was apparent a single herd was passing the camera. We classified conservation status of mammals following the IUCN Red List of Threatened Species (IUCN, 2022) and National Mammals Red List of Nepal (Jnawali et al., 2011).

### 2.3 Data analysis

We filtered, arranged, and summarized the data using the package tidyverse (Wickham & Wickham, 2017) in R Program (R Core Team, 2022), then created a detection matrix with the number of detections for each species across occasions. Before analysis, we performed the correlation and collinearity analysis to identify whether the independent variables were highly related or not. The analyses revealed none of the selected variables were correlated (Pearson's correlation |*r*| < .7) (Dormann et al., 2013) or co‐linear (Variation Inflation Factor [VIF] <10; O'brien, 2007).

#### 2.3.1 Hierarchical multispecies occupancy

We conducted the hierarchical analysis following Royle and Dorazio (2008) in which the Beta was replaced prior for species‐level *ψ*_k_:

$$\log\mathrm{it}\left(\psi_{k}\right)∼\text{Normal}\left(\mu_{\text{lpsi}},\sigma_{\text{lpsi}}\right)$$

and the community‐level hyperpriors were added as

$$\bar{\psi }∼\text{Beta}\left(1,1\right)$$

$$\mu_{\text{lpsi}}=\log\mathrm{it}\left(\bar{\psi }\right)$$

$$\sigma_{\text{lpsi}}∼\text{Uniform}\;\left(0,5\right)$$

We subjected the analysis to Markov Chain Monte Carlo (MCMC) simulations to obtain the posterior distributions. We ran 50,000 iterations for each of three chains then visually inspected whether the chains mixed well. We confirmed mixing using trace plot diagnostics and Rhat values. If the optimal value of Rhat was <1.1, we did a further step in analysis by adjusting the number of iterations until the Rhat became <1.05. After the successful convergence of markov chains for all parameters, the value of detection probability (*p*) and naïve occupancy (*ψ*) including mean and standard deviation (SD) of *p* and *ψ* were obtained.

#### 2.3.2 Effect of covariates

As occupancy probability varies across sites, we calculated occupancy for each species *k* at site *i* using the following formula:

$$\text{logit}\;\left(\psi_{\mathrm{ik}}\right)=\beta_{0,k}+\beta_{\mathrm{cov},k}{\mathrm{cov}}_{i},$$

where the coefficients (*β*) differ among species. We used species level priors as:

$$\beta_{x,k}∼\text{Normal}\;\left(\mu_{x},\sigma_{x}\right),$$

where *μ*_x_ and *σ*_x_ are the coefficients of random variable drawn from a normal distribution with mean and SD to be estimated. Unlike previous models, we incorporated correlation between *ψ* and *ρ*, where the intercept *β*_0,*k*_, is the probability of occupancy of species *k* at a site with a given combinations of variables (Devarajan et al., 2020).

For community‐level hyperpriors, we used a uniform Beta (1,1) prior to the probability and converted it to the logit scale where: ${\bar{B}}_{0}\sim \text{Beta}\;\left(1,1\right)$ and $\mu_{0}=\log\mathrm{it}\;\left({\bar{\beta }}_{0}\right)$. We used a uniform prior for the SD as: Σ_0_ ~ Uniform (0,5). The coefficient value at logit scale will be around ±5, therefore, *μ*_x_ = Uniform (−5, 5) and the SD is *σ*_x_ = Uniform (0,5). For hierarchical analysis, we used an adaptive MCMC with 50,000 iterations, three chains, 1000 adaptations, and a burn in of 1000. We performed occupancy analyses using Just Another Gibbs Sampler (Plummer, 2003) and R Program (R Core Team, 2022) using packages coda (Plummer et al., 2006) and jagsUI (Kellner et al., 2019).

## 3 RESULTS

We detected 15 mammal species (83 total detections) from eight families and four orders in 1530 camera days. One species (red panda) is categorized as endangered, four species as vulnerable, one near threatened, and nine as least concern in the IUCN Red List of Threatened Species (IUCN, 2022). Using the national red list of Nepal 2011 (Jnawali et al., 2011), two species were under the category endangered, three vulnerable, one near threatened, six were least concerned, and remaining three were data deficient. The most detected species was red fox (*Vulpes vulpes*; 21 times across 16 sites), while the least observed species were blue sheep and Jungle cat (*Felis chaus*) which were detected once each.

### 3.1 Hierarchial multispecies occupancy analysis

**FIGURE 2:**
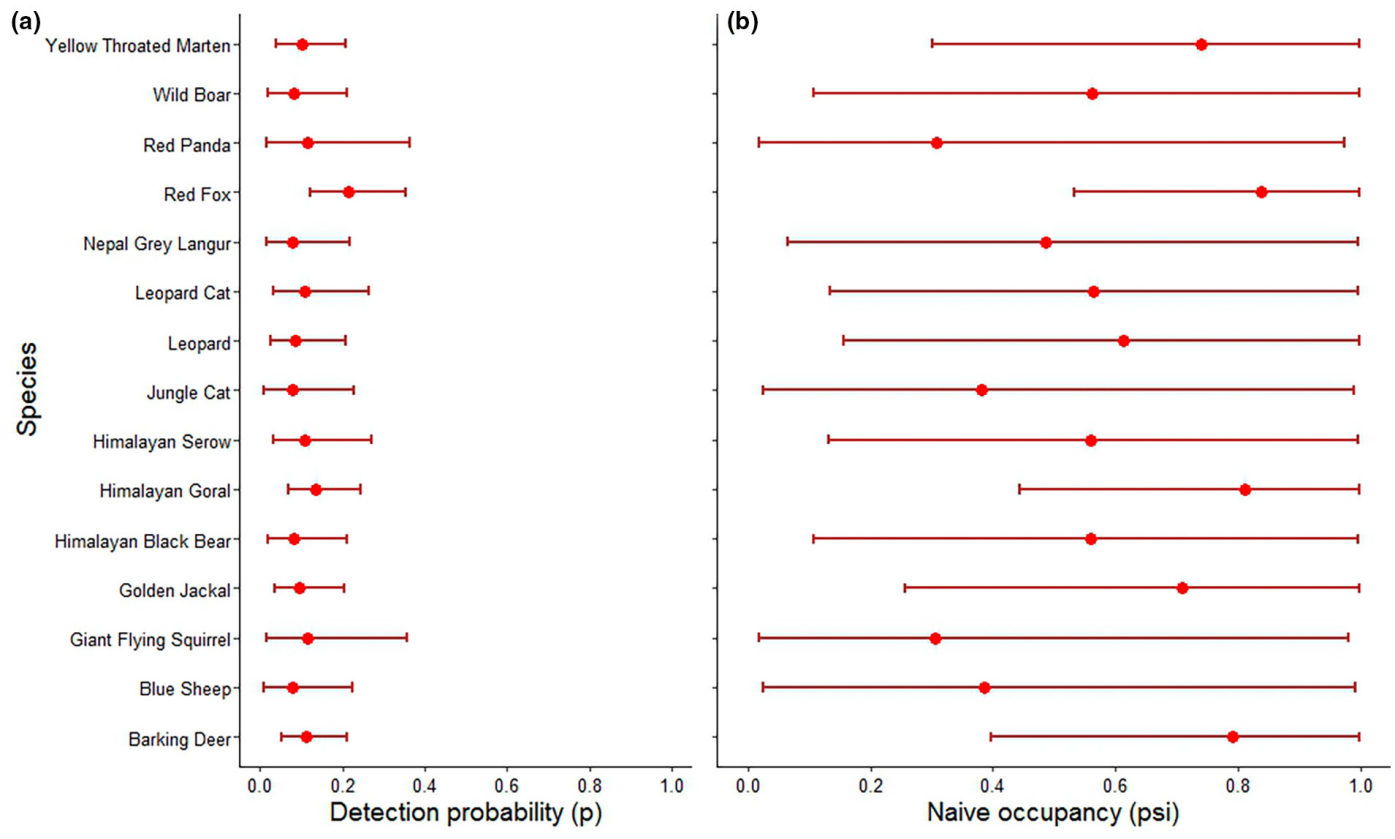
(a) Detection probability (*p*) and (b) Naïve occupancy (*ψ*) for each of the 15 mammal species across five replicate surveys in Dhorpotan Hunting Reserve, Nepal, 2022.

We found good convergence between the MCMC chains for posterior distributions during the analysis of Bayesian inference. Rhat values were <1.05 for all parameters; meancommunity‐level detection probability and mean naïve occupancy were 0.097 ± 0.036 and 0.600 ± 0.20, respectively (Table 1; Figure 2). Detection probabilities ranged from 0.079 to 0.210, suggesting community composition was comparatively heterogeneous. The highest detection probabilities were for red fox (*p* = .210 ± .050) and red panda (*p* = .120 ± .090), whereas lowest detection probabilities were for blue sheep (*p* = .079 ± .061), Nepal gray langur (*p* = .080 ± .056), and jungle cat (*p* = .080 ± .062). We observed a modest detection probability of wild boar (*p* = .083 ± .053). Naïve occupancy ranged from 0.310 to 0.838, suggesting probability of occupancy varied among sites and occasions. We observed highest naïve occupancy for red fox (*ψ* = 0.838 ± 0.135), followed by Himalayan goral (*ψ* = 0.810 ± 0.160) and barking deer (*ψ* = 0.791 ± 0.176). For wild boar, the naïve occupancy was (0.561 ± 0.282). The lowest naïve occupancy (*ψ* = 0.310 ± 0.250) was observed for Hodgson's giant‐flying squirrel and red panda.

**TABLE 1:** Community‐level detection probability (*p*) and naïve occupancy (*ψ*) of mammalian community in Dhorpatan Hunting Reserve, Nepal, 2022.

Parameters	Mu	SD	LCI	Md	UCI	Rhat	ESS	Overlap0	*F*
*p*	0.097	0.036	0.027	0.050	0.183	1	36,642	0	1
*ψ*	0.600	0.200	0.025	0.501	0.975	1	138,772	0	1

*Note*: Mean (mu), standard deviation (SD), lower confidence interval (LCI), median (md), upper confidence interval (UCI), Rhat, effective sample size (ESS), overlap0 (proportion of posterior with same size), and *f* statistics (*F*) of the distribution.

### 3.2 Effects of covariates

**FIGURE 3:**
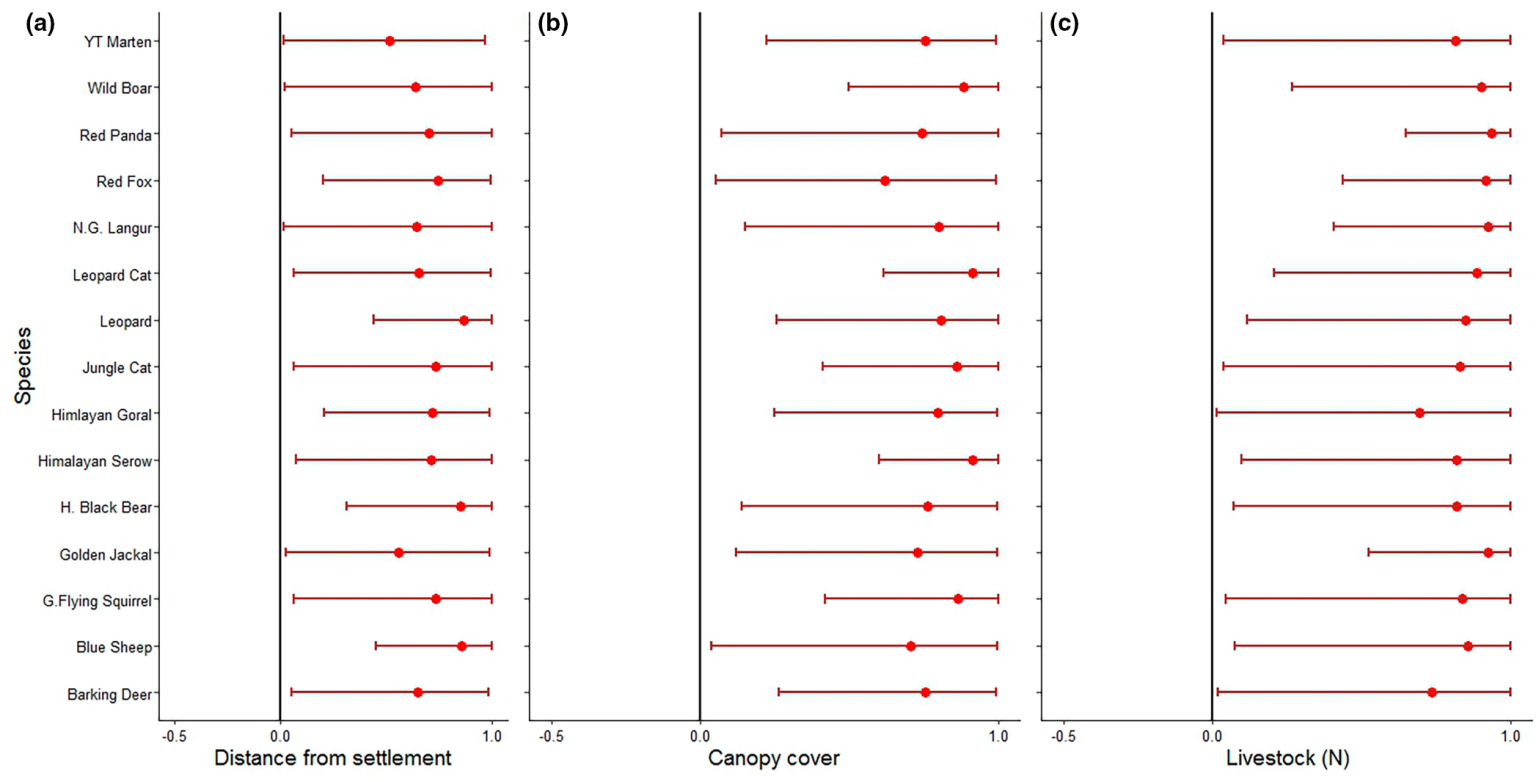
Caterpillar plots showing the effect size of the covariates on each mammal species detected, Dhorpotan Hunting Reserve, Nepal, 2022.

Mean canopy cover across sites was 21.1 ± 22.2%. The average distance to nearest human settlement was 1592.9 ± 1205.2 (SD) m and the average number of times livestock were detected during the study was 24.8 ± 47.3 (SD). Mammal community occupancy increased with increasing number of livestock detections (3.104 ± 1.304) and increasing canopy cover (2.158 ± 1.107) and Bayesian credible intervals did not overlap 0 (Table 2; Figure 3). Mammal community occupancy appeared to decrease when closer to human settlements (1.451 ± 1.107) but the effect was not significant.

**TABLE 2:** Community‐level summaries of the hyperparameters for occupancy hypothesized to influence the occupancy probabilities of mammalian community in Dhorpotan Hunting Reserve, Nepal, 2022.

Covariates	Mu	SD	LCI	Md	UCI	Rhat	ESS	*O*	*F*
b0 (intercept)	0.710	0.189	0.275	0.745	0.970	1	5565	0	1
Distance (settlement)	1.451	1.107	−0.510	1.361	3.914	1.001	2461	1	0.925
Canopy cover	2.158	1.107	0.287	2.047	4.521	1.002	1203	0	0.989
Livestock detected (no.)	3.104	1.304	0.219	3.306	4.916	1.004	747	0	0.982

*Note*: Mean (mu), standard deviation (SD), lower confidence interval (LCI), Median (md), upper confidence interval (UCI), Rhat, effective sample size (ESS), overlap (O), and proportion of posterior with same size as mean (F).

**FIGURE 4:**
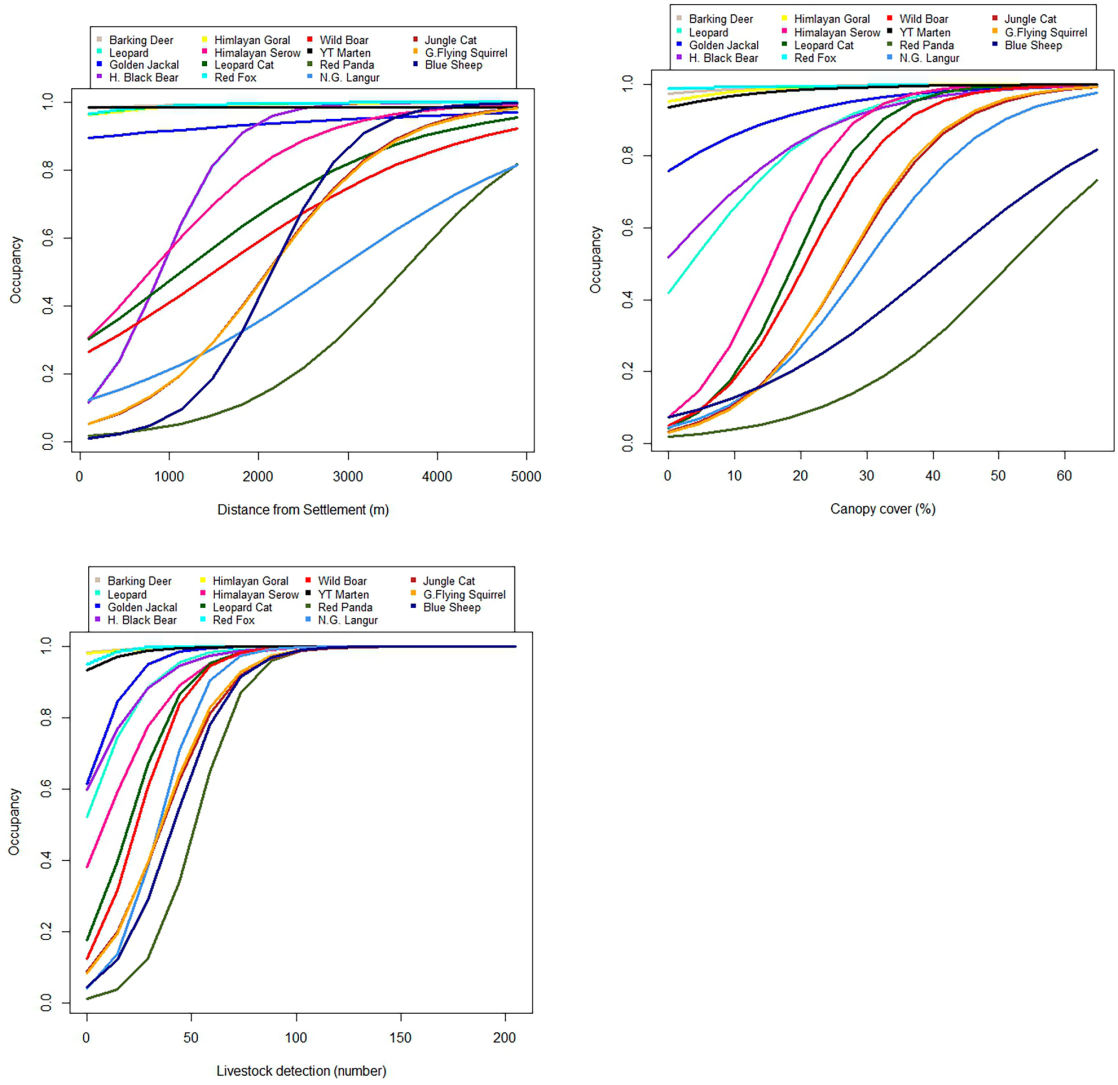
Effects of covariates number of livestock detected, canopy cover, and distance from human settlement on the occupancy of 15 mammal species, Dhorpatan Hunting Reserve, Nepal, 2022.

The occupancy of all species increased with increasing livestock detections except for Himalayan goral (intercept = 1.70 ± 2.95) which remained relatively constant (Figure 4). The greatest increase in occupancy with number of livestock detections was observed for red panda (intercept = 4.14 ± 2.50). We observed a positive impact of livestock detection on occupancy of wild boar (intercept = 3.88 ± 2.735). Occupancy of all species increased with the increase in canopy cover, with greatest increase observed for Himalayan serow (3.70 ± 2.42), leopard cat (intercept = 3.65 ± 2.34), and wild boar (3.00 ± 2.28). Red fox occupancy (intercept = 0.76 ± 1.87) appeared unaffected by canopy cover. Occupancy of most species increased with increasing distance from human settlement, especially for Himalayan black bear (3.08 ± 2.60) and leopard (3.05 ± 2.39). In case of wild boar, however, only a slight increase in occupancy was observed (intercept = 0.88 ± 2.32). In contrast, a slight decline in occupancy of Yellow‐throated marten (intercept = 0.01 ± 1.85) was observed as distance from settlements increased.

## 4 DISCUSSION

The study confirmed the heterogenous mammal species composition in the study area and anthropogenic effects such as livestock presence on their habitat is rampant. Basically, we found overall avoidance of humans (i.e., settlements), with mammals selecting for greater forest cover. It means the anthropogenic activities are altering (largely negatively) mammal communities. The high value of SD for the covariates means that the study plots also were highly heterogeneous in the variable composition. We also identified a lesser to no effect of the covariates on the species like red fox, barking deer, and Himalayan goral. These species are widespread species in Nepal and are more generalist in their habitat selection (Jnawali et al., 2011); therefore, no major impact of variables was observed. Number of livestock detected and canopy cover were observed to exert a positive impact on mammalian community occupancy in our study human settlement exerted a negative impact. Number of livestock detection was observed to influence the most on species occupancy positively with a sharp increase in response. It might be due to common sharing of grazing grounds between livestock and wild herbivores or presence of both prey and carnivore species in the same habitat (Kalle et al., 2013). The DHR is one of the pastureland; therefore, local people or people near to the reserve leave their livestock for grazing which might support the coexistence of wild mammal species and livestock. The highest positive impact of livestock detection was observed on red panda which might be due to sharing of bamboo as food by red panda and livestock (Sharma et al., 2014). The positive impact of herbivore on red panda presence was also reported in protected area system of Nepal (Acharya et al., 2018). The increased occupancy of wild boar with near to human settlement in this study indicates the potentiality of this species for human‐wildlife conflict in DHR as found in other protected areas (Pandey et al., 2016). However, in case of Himalayan goral, the relatively less impact of livestock presence might be it being a widespread species in the study area. The relatively low detection rate of blue sheep within the study area could potentially be attributed to a decline in population size in recent years, as well as their known preference for terrain slopes as a habitat (DHR, 2019).

The increase in forest canopy cover supports to increase in the occupancy of all species. It might be due to their preferences toward dense habitat potentially to avoid human disturbances and hiding place with predators (Laurance et al., 2008; Regolin et al., 2017; Whitworth et al., 2019). The dense vegetation provides a better hideout for prey species as well as ambushing spots for predators (Monroy‐Vilchis et al., 2009). Himalayan serow was the species most positively impacted by the percentage of canopy cover. The preference of dense vegetation covers by Himalayan serow was also observed in Kanchendzonga Biosphere Reserve, India (Bhattacharya et al., 2012), which might be due to it being prey species and preferring dense vegetation to avoid predators.

Occupancy of all species increased with the increase in distance from human settlement except for Yellow‐throated marten. The increase in occupancy of all species with the increase in distance from human settlement generally relates to the increase of anthropogenic disturbance as human settlements offer different threats to wildlife like poaching as well as guard dogs (Cavada et al., 2019; Salvatori et al., 2022; Schuette et al., 2013). The decrease in occupancy of species with proximity to the settlement due to the hunting and poaching pressure is rather common observation in case of ungulates (Soh et al., 2014). Carnivores also prefer to roam away from settlements and are observed to avoid possible human encounters to avoid poachers as well as retaliation from local farmers (Drouilly et al., 2018; Kalle et al., 2013; Pia et al., 2013). The decrease in occupancy of Yellow throated marten with increase in distance from human settlement correlates to its prey preference as it prefers small livestock prey species like avian livestock (Baral et al., 2021) and human associated small mammal prey species like rats (*Rattus rattus*; Parr & Duckworth, 2007; Zhou et al., 2008).

The occupancy of barking deer, red fox, and Himalayan goral remained relatively stable across all variables, which might be due to higher species abundance as well as the ecological aspects of the species in the study area. These species are observed to have widespread distribution range across Nepal (Jnawali et al., 2011). Higher site occupancy for Barking deer was observed in the area where low impacts of variables are noticed (Letro et al., 2022). The occupancy of red fox was also high across different habitats in Tieqiaoshan Nature Reserve, China, with less extinction probabilities in sites (Vitekere et al., 2020) as well as relatively stable impacts of associated variables (MacDougall & Sander, 2022). Himalayan goral is also one of the species with higher occupancy across the study area and was observed to be less impacted by the variables involved. This might be due to the lesser preference of elevation by the species as well as lesser selectivity of habitat by the species (Bhattacharya et al., 2012).

## 5 CONCLUSIONS

Based on our study, it is evident that there is an overall negative anthropogenic impact on mammalian occupancy in the DHR. Our findings indicate that human settlements have a significant negative impact on mammalian occupancy and that mammals prefer dense canopy. Additionally, the high level of interaction between wild mammals and livestock raises the probability of human‐associated threats to wildlife. The interactions between humans and wildlife can lead to conflicts and a negative attitude toward wildlife in the area, which can make it difficult to conserve wildlife. As such, we recommend that proper management approaches be implemented in DHR, with a particular focus on reducing the interactions between humans and wild mammal species. This can include measures such as increasing the distance between human settlements and wildlife habitats, educating the local community about wildlife conservation, and implementing measures to prevent human‐wildlife conflicts.

## AUTHOR CONTRIBUTIONS

**Sandeep Regmi:** Conceptualization (equal); data curation (equal); formal analysis (equal); funding acquisition (lead); investigation (lead); methodology (equal); writing – original draft (equal); writing – review and editing (equal). **Jerrold L. Belant:** Writing – review and editing (equal). **Bindu Pant:** Writing – review and editing (equal). **Hari Prasad Sharma:** Conceptualization (equal); data curation (equal); formal analysis (equal); methodology (equal); supervision (equal); writing – original draft (equal); writing – review and editing (equal).

## CONFLICT OF INTEREST STATEMENT

Authors declare no conflict of interest.

## FUNDING INFORMATION

University Grants Commission, Nepal (77/78‐S&T‐38).

## Data Availability

All the relevant data used in this study will be archived in Dryad https://doi.org/10.5061/dryad.6m905qg3s.
